# Intraocular lens power calculation formulas accuracy in combined phacovitrectomy: an 8-formulas comparison study

**DOI:** 10.1186/s40942-021-00315-7

**Published:** 2021-08-18

**Authors:** Diogo Hipólito-Fernandes, Maria Elisa Luís, Diogo Maleita, Pedro Gil, Vitor Maduro, Lívio Costa, Nuno Marques, João Branco, Nuno Alves

**Affiliations:** Department of Ophthalmology, Centro Hospitalar Universitário de Lisboa Central, Alameda de Santo António dos Capuchos, 1169-050 Lisbon, Portugal

**Keywords:** Vitreomacular interface disorders, Phavocitrectomy, Intraocular lens power, Formulas accuracy

## Abstract

**Background:**

Our study aimed to assess and compare the accuracy of 8 intraocular lens (IOL) power calculation formulas (Barrett Universal II, EVO 2.0, Haigis, Hoffer Q, Holladay 1, Kane and PEARL-DGS) in patients submitted to combined phacovitrectomy for vitreomacular (VM) interface disorders.

**Methods:**

Retrospective chart review study including axial-length matched patients submitted to phacoemulsification alone (Group 1) and combined phacovitrectomy (Group 2). Using optimized constants in both groups, refraction prediction error of each formula was calculated for each eye. The optimised constants from Group 1 were also applied to patients of Group 2 – Group 3. Outcome measures included the mean prediction error (ME) and its standard deviation (SD), mean (MAE) and median (MedAE) absolute errors, in diopters (D), and the percentage of eyes within ± 0.25D, ± 0.50D and ± 1.00D.

**Results:**

A total of 220 eyes were included (Group 1: 100; Group 2: 120). In Group 1, the difference in formulas absolute error was significative (p = 0.005). The Kane Formula had the lowest MAE (0.306) and MedAE (0.264). In Group 2, Kane had the overall best performance, followed by PEARL-DGS, EVO 2.0 and Barrett Universal II. The ME of all formulas in both Groups 1 and 2 were 0.000 (p = 0.934; p = 0.971, respectively). In Group 3, a statistically significant myopic shift was observed for each formula (p < 0.001).

**Conclusion:**

Surgeons must be careful regarding IOL power selection in phacovitrectomy considering the systematic myopic shift evidenced—constant optimization may help eliminating such error. Moreover, newly introduced formulas and calculation methods may help us achieving increasingly better refractive outcomes both in cataract surgery alone and phacovitrectomy.

**Supplementary Information:**

The online version contains supplementary material available at 10.1186/s40942-021-00315-7.

## Background

Phacovitrectomy, the combination of phacoemulsification, pars plana vitrectomy (PPV) and intraocular lens (IOL) implant, became increasingly popular over the past few years for the treatment of vitreoretinal disorders [1–4]. Considering that up to 80% of eyes develop nuclear sclerotic cataract within 2 years after PPV [5] and the higher complications rate of phacoemulsification in vitrectomized eyes [6], a combined procedure is being considered even in the absence of a significant cataract. Furthermore, a single surgery has a number of advantages: improved retina visualization, faster visual acuity recovery, safer vitreous shaving without concerns about intraoperative lenticular touch and lower cost [7].

Over the years, several studies reported the refractive outcomes of combined phacovitrectomy [8–18]. The achievement of a target refractive outcome became increasingly important since patient’s refractive expectations increased and due to technological advances. However, at present, IOL power calculations are performed without adjustments considering the added vitrectomy. This may account for the reported mixed results, some of them revealing a lower accuracy of refractive predictions in such patients, compared with phacoemulsification alone [13, 16].

Recently, some modern IOL lens power calculation formulas appeared, using new methodologies and a large number of preoperative eye parameters to calculate the postoperative refractive error. The Kane formula^A^ is based on theoretical optics and incorporates both regression and artificial intelligence components to refine predictions. Emmetropia Verifying Optical (EVO) formula^B^ is based on the theory of emmetropization (optimized version 2.0 has been recently released), and finally, PEARL-DGS formula^C^, the most recently available, uses machine learning modelling and output linearization to predict ELP and adjust it for extreme biometric values.

The comparison of formulas performance has been largely investigated for phacoemulsification [19–22]. However, there is a lack of literature regarding phacovitrectomy. To the best of our knowledge, there is only one recently published article comparing formulas accuracy in phacovitrectomized eyes [16]. Moreover, we are unaware of comparative studies using an optical low-coherence reflectometry (OLCR) biometer and including PEARL-DGS and EVO 2.0 formulas.

Our study aims to assess the overall accuracy of three new-generation and five vergence-based formulas in patients submitted to combined phacovitrectomy for vitreomacular (VM) interface disorders, using measurements obtained from an OLCR biometer (Lenstar LS900^®^). The impact of constant optimization on refractive outcomes will also be investigated.

## Methods

### Patients and surgical procedure

Retrospective chart review study, including patients who were submitted to phacovitrectomy or phacoemulsification alone, between 2017 and 2019. The study adhered to the principles of the Declaration of Helsinki and was approved by the institution ethics committee.

Eligible patients were organized into two groups: Group 1: uneventful phacoemulsification with spherical hydrophobic monofocal in-the-bag IOL insertion; Group 2: uneventful combined phacovitrectomy, with spherical hydrophobic monofocal in-the-bag IOL insertion.

Exclusion criteria were: (1) incomplete biometry data; (2) postoperative corrected distance visual acuity inferior to 20/40; (3) keratometric astigmatism > 4D; (4) any intraoperative or postoperative complication; (5) previous intraocular or refractive surgery; (6) previous ocular trauma; (7) any corneal disease, as keratoconus or corneal scarring of any etiology; (7) secondary epiretinal membranes or full-thickness macular holes; (8) other retinal disease. Phacovitrectomies were considered for symptomatic patients (decreased visual acuity or metamorphopsia), due to any vitreomacular interface disease (epiretinal membrane, vitreomacular traction and full-thickness macular hole).

All surgeries were performed by two experienced surgeons (NM and JB), with a similar proportion of surgeries performed by each surgeon in both groups. In all cases, a 2.75 mm clear corneal incision was used for phacoemulsification, with subsequent intraocular lens insertion—AcrySoft IQ SN60WF (Alcon Laboratories, Geneva, Switzerland). In Group 2, 23-gauge vitrectomy, including core vitrectomy, posterior vitrectomy, and vitreous base shaving, was performed. Brilliant blue dye and a 23-gauge Grieshaber Revolution^®^ DSP forceps (Alcon Laboratories, Geneva, Switzerland) was used to peel the internal limiting membrane and, in selected cases, 20% sulfur hexafluoride (SF6) gas tamponade was used.

According to Hoffer et al. [23] recommendations, only one eye per patient was included. If both eyes of the same patient fulfilled the above-mentioned criteria, the eye included was chosen randomly. Only eyes with an axial length (AL) between 21.0 mm and 27.0 mm were included—groups were matches according to the AL, with Group 1 being considered as the control for Group 2.

Preoperatively, patients were submitted to a complete ophthalmological examination, including optical coherence tomography (OCT) of the posterior segment (Spectralis^®^, Heidelberg Engineering, Germany), to determine macular thickness (the Early Treatment Diabetic Retinopathy Study map was used to evaluate macular thickness in the central subfield region before surgery). Optical biometry was performed with optical low-coherence reflectometry (OLCR)—Lenstar LS-900 (Haag-Streit AG, Köniz, Switzerland), obtaining the following data for each patient: AL, anterior chamber depth (ACD), central corneal thickness (CT), keratometry (K), lens thickness (LT) and horizontal corneal diameter. Postoperative manifest refraction was assessed 8 weeks after the surgery by an ophthalmologist.

### Formula calculation

For both groups (1 and 2), spherical equivalent predictions from eight IOL power calculation formulas were obtained, using a keratometer index of 1.3375. The SRK/T [24], Holladay 1, [25] Haigis [26] and Hoffer Q [27] formulas were calculated using the newly released platform IOLzero^©D^. The results were confirmed and validated against optical biometer printouts by one of the authors (DHF). Barrett Universal II (Barrett UII), Kane, EVO 2.0 and PEARL were calculated through their respective publicly-available websites^A,B,C,E^.

Each formula constant used was optimized for each group, to achieve an arithmetic mean prediction error (ME) of zero, according to protocols [23]. The Haigis formula underwent single optimization using ULIB (User Group for Laser Interference Biometry) constants for a1 and a2, as suggested by Melles et al. [28].

Any small residual ME was nulled by adjusting the refractive prediction error for each eye up or down by an amount equal to the ME of that group, as described by Wang et al. [29].

### Outcome measurements

Refractive prediction error, the primary outcome, was calculated as the difference between the spherical equivalent of the post-operative manifest refraction and the formula prediction error. A negative refractive prediction error means a myopic result and a positive prediction error represents a hyperopic outcome [29].

Study outcome measures included the ME and its standard deviation (SD), the mean absolute error (MAE) and median absolute prediction error (MedAE) of each formula, following Wang et al. [29] recommendations. The percentage of eyes with a prediction error within ± 0.25, ± 0.50 and ± 1.00 diopters were also calculated. The optimized constants of the phacoemulsification group (Group 1) were applied in the phacovitrectomy group (Group 2), and the newly generated refractive predictions errors of each formula were calculated (Group 3). Finally, formulas were ranked according to Cooke et al. guidelines [30].

### Statistical analysis

Demographics and biometric data of patients were described with frequencies (percentages) and mean (SD: standard deviation). Data normality was assessed by the Kolmogorov–Smirnov test. Parametric independent samples *t-*test, nonparametric Mann–Whitney-*U* test and chi-square test were applied, as appropriate, for group comparisons. Parametric one sample t-test or nonparametric Wilcoxon signed-rank test (1 sample) were used, as appropriate, to evaluate whether the mean refractive prediction error of each formula was different from zero. ANOVA with repeated measures were used to compare formulas prediction error in each group. The comparisons of the absolute errors were assessed using the Friedman test (nonparametric ANOVA) with Bonferroni correction, as recommended [29, 31], using Dunn post-test. The Cochran Q test was used to compare the percentage of eyes within ± 0.25D, ± 0.50D and ± 1.00D, with Bonferroni adjustment, using Dunn post-test. A level of significance α = 0.05 was considered. Statistical analysis was performed using SPSS for Windows Software (version 24.0, SPSS, Inc).

For sample size calculation, the PS program (version 3.0.12; Dupont WD, Plummer WD Jr. PS: Power and Sample Size Calculation, version 3.0. Department of Biostatistics, Vanderbilt University, Nashville, USA, 2012) was used. It was estimated that a sample size of 97 eyes would be necessary to identify a difference of one third of the standard deviation of differences in refractive prediction error, with a significance level of 5% and a test power of 90%.

## Results

### Demographics and biometric data

A total of 220 eyes of 220 caucasian patients were included (Group 1: 100; Group 2: 120). Of the 120 phacovitrectomized eyes, 85 had the diagnosis of epiretinal membrane, 26 of full-thickness macular hole and 9 of vitreomacular traction (mean preoperative macular thickness was 473 ± 77 µm, 364 ± 82 µm and 381 ± 91 µm, respectively). SF6 gas tamponade was used in forty-two (35%) patients. Patients’ biometric data, by group, are presented in Table 1.

**Table 1:** Demographic and biometric data of patients, by group

Parameter	Group 1 Phacoemulsification alone n = 100	Group 2 Combined phacovitrectomy n = 120	p-value
Age, years	76.72 ± 10.34 (46–94)	74.35 ± 6.51 (59–89)	0.001^1^
Female gender, n (%)	52 (52.0)	52 (42.3)	0.218^2^
Axial length, mm	23.52 ± 1.13 (21.14–26.91)	23.41 ± 0.99 (21.26–26.91)	0.320^1^
Anterior chamber depth, mm	3.23 ± 0.43 (2.06–4.64)	3.21 ± 0.41 (2.33–4.32)	0.762^3^
Mean Keratometry, D	43.92 ± 1.55 (40.69–47.96)	44.14 ± 1.40 (40.28–47.27)	0.211^3^
Corneal Thickness, µm	538.76 ± 32.71 (472–645)	548.39 ± 33.58 (457–654)	0.694^3^
Lens Thickness, mm	4.39 ± 0.51 (2.56–5.44)	4.52 ± 0.45 (3.59–5.79)	0.321^3^
Horizontal corneal diameter, mm	11.95 ± 0.42 (10.99–12.82)	11.86 ± 0.44 (10.41–13.27)	0.722^3^
Implanted IOL power, D	21.39 ± 2.87 (12.0–28.0)	21.27 ± 2.69 (12.0–29.0)	0.739^1^
Postoperative refractive SE, D	− 0.06 ± 0.55 (− 2.13 to 1.63)	− 0.06 ± 0.58 (− 1.38 to 1.38)	0.860^1^

Results are expressed as mean ± standard deviation (range)

*IOL* intraocular lens, *SE* spherical equivalent, *D* diopters

^1^Mann-Whiney-U test; ^2^Chi-square test; ^3^Independent samples *t*-test

There was no statistically significant difference regarding biometric measurements between Group 1 and Group 2. Optimised constants are shown in Additional file 1: Table S1.

### Formulas accuracy

Tables 2, 3 and 4 reveal the outcomes of each formula in Group 1, 2 and 3, respectively.

**Table 2:** Group 1 (phacoemulsification alone) overall outcomes of each formula

Formula	ME	SD	MAE	MedAE	Percentage of eyes within			Rank
					± 0.25D	± 0.50D	± 1.00D	
Kane	0.000	0.383	0.306	0.264	46.4	81.4	99.0	1.2
PEARL-DGS	0.000	0.385	0.312	0.282	46.5	81.4	99.0	2.0
Barrett UII	0.000	0.398	0.326	0.288	42.3	85.6	99.0	2.7
EVO 2.0	0.000	0.391	0.313	0.271	45.4	80.4	99.0	3.2
Holladay 1	0.000	0.410	0.336	0.293	45.4	74.2	99.0	3.3
Haigis	0.000	0.420	0.344	0.320	41.2	76.3	97.9	4.7
Hoffer Q	0.000	0.435	0.357	0.312	39.2	73.2	99.0	5.0
SRK/T	0.000	0.436	0.363	0.328	35.1	74.2	97.9	6.5

*ME* mean prediction error, *SD* standard deviation, *MAE* mean absolute error, *MedAE* median absolute error

**Table 3:** Group 2 (combined phacovitrectomy) overall outcomes of each formula

Formula	ME	SD	MAE	MedAE	Percentage of eyes within			Rank
					± 0.25D	± 0.50D	± 1.00D	
Kane	0.000	0.471	0.364	0.270	48.3	71.6	95.7	1.7
PEARL-DGS	0.000	0.486	0.377	0.295	44.8	74.1	95.7	1.8
EVO 2.0	0.000	0.485	0.375	0.308	41.4	72.4	94.0	2.5
Barrett UII	0.000	0.508	0.393	0.288	41.4	69.0	96.6	3.5
Haigis	0.000	0.525	0.392	0.315	40.5	71.6	90.5	4.8
Holladay 1	0.000	0.532	0.406	0.311	42.2	70.7	91.4	4.8
SRK/T	0.000	0.532	0.411	0.319	37.1	66.4	93.1	5.7
Hoffer Q	0.000	0.554	0.419	0.329	38.8	69.8	91.4	6.0

*ME* mean prediction error, *SD* standard deviation, *MAE* mean absolute error, *MedAE* median absolute error

**Table 4:** Group 3 (combined phacovitrectomy—group 2—eyes using group 1 optimised constants) overall outcomes of each formula

Formula	ME	SD	MAE	MedAE	Percentage of eyes within			Rank
					± 0.25D	± 0.50D	± 1.00D	
Kane	− 0.140	0.472	0.374	0.315	46.6	68.1	95.7	2.8
Barrett UII	− 0.137	0.509	0.402	0.293	43.1	69.8	94.8	3.2
PEARL-DGS	− 0.169	0.489	0.396	0.293	44.8	69.8	93.1	3.3
EVO 2.0	− 0.151	0.491	0.393	0.277	47.4	69.0	94.8	3.7
Haigis	− 0.126	0.528	0.403	0.290	46.6	70.7	91.4	4.5
Hoffer Q	− 0.133	0.562	0.417	0.270	38.8	69.8	92.2	5.0
Holladay 1	− 0.132	0.532	0.403	0.280	46.6	68.1	92.2	5.2
SRK/T	− 0.155	0.533	0.423	0.335	41.4	69.0	91.4	6.8

*ME* mean prediction error, *SD* standard deviation, *MAE* mean absolute error, *MedAE* median absolute error

There was no statistically significant difference between formulas ME in both groups 1 and 2, since they were 0.000D for all formulas (p = 0.934; p = 0.971, respectively).

In the phacoemulsification group (Group 1), the Kane formula had the overall best performance (lowest SD, MAE and MedAE), but it was the Barret Universal II who presented the highest percentage of eyes within ± 0.50D (85.6%). There was a significant difference between formulas’ absolute errors (p = 0.005). After post-hoc analysis, the Kane formula performed better than the SRK/T (p = 0.03). The proportion of eyes within ± 0.50D was also significantly different among evaluated formulas (p = 0.03) but after Bonferroni correction there was no evidence of superiority of any formula.

Regarding phacovitrectomy group (Group 2), there was no statistically significant difference between formulas’ absolute errors and percentage of eyes within a certain prediction error (± 0.25D, ± 0.50D and ± 1.00D). Overall, the Kane formula had the lowest SD, MAE, MedAE and the highest percentage of eyes within ± 0.25D. The PEARL-DGS formula presented the highest percentage of eyes within ± 0.50D (74.1%). Both formulas had 95.7% of eyes within ± 1.00D.

In Group 3 (phacovitrectomy patients using Group 1 optimized constants), the Kane formula presented the lowest SD and MAE, even without a statistically significant difference. The Haigis formula had the highest percentage of eyes within ± 0.50D (70.7%). The formula which presented the lower percentage of refractive surprises (prediction error superior to 1.00D) was Kane (4.3%). In this group, the ME of all formulas was significantly different from zero (p < 0.001 for each formula), with a tendency for a myopic shift. Comparing groups 2 and 3, there was a statistically significant difference between formulas ME (p < 0.05 for each of the analysed formulas) but no difference was found with respect to the MAE (p > 0.05 for each formula). No statistically significant difference was found in any of the analysed formulas regarding the comparison of the ME in Group 3 between patients with and without SF6 gas tamponade (p > 0.05 for each formula).

Globally, irrespective of the group, the Kane formula had consistently the most accurate performance (lowest MAE and best rank) of all formulas, followed by the other multiple-parameter formulas (PEARL-DGS, EVO 2.0 and Barrett UII).

## Discussion

Our study evaluated the accuracy of new generation formulas such as Kane, EVO 2.0 and PEARL-DGS, along with five well validated vergence-based formulas (Barrett UII, SRK/T, Holladay 1, Hoffer Q and Haigis) both in patients submitted to phacoemulsification alone or combined phacovitrectomy.

Reported refractive outcomes revealed good results after phacovitrectomy, frequently comparable with the outcomes obtained with phacoemulsification [10, 14, 17, 18]. However, there is a lack of literature reporting formulas accuracy following the strict recommendations for IOL power formulas studies [23, 31]. To the best of our knowledge this is just the second clinical study following those recommendations in phacovitrectomized eyes. Furthermore, it is the first analysing some of these new formulas (EVO 2.0 and PEARL-DGS) and using data obtained from an OLCR-biometer (Lenstar LS900).

Overall, we revealed that in the axial-length matched control group (Group 1: phacoemulsification alone), multiple-parameter formulas, which include Kane, PEARL-DGS, EVO 2.0 and Barrett UII performed better, with the Kane formula having the best rank among all. These results agree with the most recent publications [19–21, 32, 33]. Moreover, all these four formulas presented a percentage of eyes within ± 0.50D superior to 80% which is comparable to the results obtained by Melles et al. study [21], in which the same OLCR-biometer was used.

Regarding Group 2 (combined phacovitrectomy), despite the absence of a statistically significant difference between the formulas, we also observed a tendency for a superiority of Kane, EVO 2.0, PEARL-DGS and Barrett UII, compared with older vergence-based. Once again, Kane formula had the best results (best rank, lowest SD, MAE, MedAE and highest percentage of eyes within ± 0.25D), agreeing with Vounotrypidis et al. [16] study.

Comparing Group 1 and Group 2, we may conclude that even using optimized lens constants in phacovitrectomized eyes, the refractive outcomes are still worse than with phacoemulsification alone: every formula had a higher SD, MAE and MedAE and a lower percentage of eyes within ± 0.50D in group 2.

Group 3 (combined phacovitrectomy using the constant optimized for the phacoemulsification group) intended to simulate a clinical practice scenario since biometers software are not able to select different constants according to different surgical procedures and optimized constants used are based on phacoemulsification alone. Multiple parameter formulas presented better ranks, particularly due to a lower SD, MAE, and percentage of refractive surprises (prediction error superior to 1.00D). These results revealed, as stated by Kane et al. [34], that these formulas tend to have a lesser number of large deviations from the expected refractive outcome, which represents the worst results that can occur.

Furthermore, we observed a consistent myopic shift for every formula (from − 0.126 up to − 0.169), which agrees with recently reported by Vounotrypidis et al. [16], who used a different biometer technology (swept-source OCT). Previous studies also showed different degrees of myopic shift after phacovictrectomy [9, 10, 13, 14]. Nonetheless, the reasons justifying it are still debatable, particularly the possible role of gas tamponade. Some authors postulated that intraocular gas tamponade induced a more anterior position of the IOL due to the buoyant effect [35] but, on the other hand, gas could induce more zonular elasticity, leading to a more posterior IOL location with an expected hyperopic shift rather than myopic [15]. There are other studies which did not find any influence of gas tamponade usage on the refractive results [10, 17, 36]. Our data corroborate those studies, revealing no difference in each formula prediction error between patients with and without SF6 gas tamponade. Other possible reasons for the reported myopic shift may be related to errors regarding axial length measurements or changes in vitreous cavity properties after vitreous removal—further studies are needed to evaluate possible related and causative factors.

Our study limitations include its retrospective design (as it is true for most studies related to IOL formulas assessment). The inclusion of data from two different surgeons may also introduce bias. However, it allows us to more accurately represent a real-world scenario and support a greater generalization of the results compared with single-surgeon studies.

Considering our study strengths, only one IOL model (AcrySoft SN60WF) was used, each surgeon performed both procedures in each case, a matched control group was used to compare the outcomes and only eyes with an AL between 21.0 mm and 27.0 mm were included, avoiding bias associated with extreme axial lengths. Besides, this study followed the strict criteria for IOL formula studies [23, 31]. We also decided to include different vitreomacular disorders because previous studies did not reveal an impact on the refractive results among them [10, 17]. Additionally, it better represents day-to-day clinical practice, in which surgeons do not use different IOL constants for different VM disorders.

Reviewing our data, we recommend that surgeons be careful regarding IOL power selection in phacovitrectomy due to the systematic myopic shift induced. Nonetheless, despite being statistically significant, we may argue if the magnitude of this shift is clinically relevant or not. We also conclude that newly introduced formulas and calculation methods may help us to achieve increasingly better refractive outcomes both in cataract surgery alone and in phacovitrectomy. The Kane formula deserves a special highlight since it was consistently the formula with the best rank, independently of the analysed group. We consider that in the future such new and more accurate formulas should be included in biometers software to avoid transcription errors and allow improved refractive results. Moreover, it would be of great interest if those softwares become able to automatically select different optimized constants according to the surgical procedure, allowing surgeons to achieve more accurate outcomes.

## Supplementary Information

**Additional file 1**: **Table S1.** Each formula optimised constants.

## Data Availability

Data are available on reasonable request, from Diogo Hipólito-Fernandes, MD (cdiogo777@gmail.com).

## References

[1] Steel DHW (2007). Phacovitrectomy: expanding indications. J Cataract Refract Surg.

[2] Scharwey K, Pavlovic S, Jacobi KW (1999). Combined clear corneal phacoemulsification, vitreoretinal surgery, and intraocular lens implantation. J Cataract Refract Surg.

[3] Senn P, Schipper I, Perren B (1995). Combined pars plana vitrectomy, phacoemulsification, and intraocular lens implantation in the capsular bag: a comparison to vitrectomy and subsequent cataract surgery as a two-step procedure. Ophthalmic Surg Lasers.

[4] Demetriades A-M, Gottsch JD, Thomsen R (2003). Combined phacoemulsification, intraocular lens implantation, and vitrectomy for eyes with coexisting cataract and vitreoretinal pathology. Am J Ophthalmol.

[5] Iwase T, Sugiyama K (2006). Investigation of the stability of one-piece acrylic intraocular lenses in cataract surgery and in combined vitrectomy surgery. Br J Ophthalmol.

[6] Cole CJ, Charteris DG (2009). Cataract extraction after retinal detachment repair by vitrectomy: visual outcome and complications. Eye.

[7] Seider MI, Michael Lahey J, Fellenbaum PS (2014). Cost of phacovitrectomy versus vitrectomy and sequential phacoemulsification. Retina.

[8] Tranos PG, Allan B, Balidis M (2020). Comparison of postoperative refractive outcome in eyes undergoing combined phacovitrectomy vs cataract surgery following vitrectomy. Graefe’s Arch Clin Exp Ophthalmol.

[9] Rahman R, Bong CX, Stephenson J (2014). Accuracy of intraocular lens power estimation in eyes having phacovitrectomy for rhegmatogenous retinal detachment. Retina.

[10] Manvikar SR, Allen D, Steel DHW (2009). Optical biometry in combined phacovitrectomy. J Cataract Refract Surg.

[11] Shiraki N, Wakabayashi T, Sakaguchi H, Nishida K (2018). Optical biometry-based intraocular lens calculation and refractive outcomes after phacovitrectomy for rhegmatogenous retinal detachment and epiretinal membrane. Sci Rep.

[12] Shi L, Chang JS, Suh LH, Chang S (2019). Differences in refractive outcomes between phacoemulsification for cataract alone and combined phacoemulsification and vitrectomy for epiretinal membrane. Retina.

[13] Hötte GJ, de Bruyn DP, de Hoog J (2018). Post-operative refractive prediction error after phacovitrectomy: a retrospective study. Ophthalmol Ther.

[14] Hamoudi H, Correll Christensen U, La Cour M (2018). Epiretinal membrane surgery: an analysis of 2-step sequential- or combined phacovitrectomy surgery on refraction and macular anatomy in a prospective trial. Acta Ophthalmol.

[15] Falkner-Radler CI, Benesch T, Binder S (2008). Accuracy of preoperative biometry in vitrectomy combined with cataract surgery for patients with epiretinal membranes and macular holes. Results of a prospective controlled clinical trial. J Cataract Refract Surg.

[16] Vounotrypidis E, Shajari M, Muth DR (2020). Refractive outcomes of 8 biometric formulas in combined phacovitrectomy with internal limiting membrane peeling for epiretinal membrane. J Cataract Refract Surg.

[17] van der Geest LJ, Siemerink MJ, Mura M, Mourits MP, Lapid-Gortzak R (2016). Refractive outcomes after phacovitrectomy surgery. J Cataract Refract Surg.

[18] Ercan ZE, Akkoyun İ, Yaman Pınarcı E, Yılmaz G, Topçu H (2017). Refractive outcome comparison between vitreomacular interface disorders after phacovitrectomy. J Cataract Refract Surg.

[19] Darcy K, Gunn D, Tavassoli S, Sparrow J, Kane JX (2020). Assessment of the accuracy of new and updated intraocular lens power calculation formulas in 10 930 eyes from the UK National Health Service. J Cataract Refract Surg.

[20] Cheng H, Kane JX, Liu L, Li J, Cheng B, Wu M (2020). Refractive predictability using the IOLMaster 700 and artificial intelligence-based IOL power formulas compared to standard formulas. J Refract Surg.

[21] Melles RB, Kane JX, Olsen T, Chang WJ (2019). Update on intraocular lens calculation formulas. Ophthalmology.

[22] Connell BJ, Kane JX (2019). Comparison of the Kane formula with existing formulas for intraocular lens power selection. BMJ Open Ophthalmol.

[23] Hoffer KJ, Aramberri J, Haigis W (2015). Protocols for studies of intraocular lens formula accuracy. Am J Ophthalmol.

[24] Retzlaff JA, Sanders DR, Kraff MC (1990). Development of the SRK/T intraocular lens implant power calculation formula. J Cataract Refract Surg.

[25] Holladay JT, Musgrove KH, Prager TC, Lewis JW, Chandler TY, Ruiz RS (1988). A three-part system for refining intraocular lens power calculations. J Cataract Refract Surg.

[26] Haigis W, Lege B, Miller N, Schneider B (2000). Comparison of immersion ultrasound biometry and partial coherence interferometry for intraocular lens calculation according to Haigis. Graefe’s Arch Clin Exp Ophthalmol.

[27] Hoffer KJ (1993). The Hoffer Q formula: a comparison of theoretic and regression formulas. J Cataract Refract Surg.

[28] Melles RB, Holladay JT, Chang WJ (2018). Accuracy of intraocular lens calculation formulas. Ophthalmology.

[29] Wang L, Koch DD, Hill W, Abulafia A (2017). Pursuing perfection in intraocular lens calculations: III. Criteria for analyzing outcomes. J Cataract Refract Surg.

[30] Cooke DL, Cooke TL (2016). Comparison of 9 intraocular lens power calculation formulas. J Cataract Refract Surg.

[31] Aristodemou P, Knox Cartwright NE, Sparrow JM, Johnston RL (2015). Statistical analysis for studies of intraocular lens formula accuracy. Am J Ophthalmol.

[32] Savini G, Di Maita M, Hoffer KJ (2020). Comparison of 13 formulas for IOL power calculation with measurements from partial coherence interferometry. Br J Ophthalmol.

[33] Savini G, Hoffer KJ, Balducci N, Barboni P, Schiano-Lomoriello D (2020). Comparison of formula accuracy for intraocular lens power calculation based on measurements by a swept-source optical coherence tomography optical biometer. J Cataract Refract Surg.

[34] Kane JX, Van Heerden A, Atik A, Petsoglou C (2016). Intraocular lens power formula accuracy: comparison of 7 formulas. J Cataract Refract Surg.

[35] Patel D, Rahman R, Kumarasamy M (2007). Accuracy of intraocular lens power estimation in eyes having phacovitrectomy for macular holes. J Cataract Refract Surg.

[36] Jeoung JW, Chung H, Yu HG (2007). Factors influencing refractive outcomes after combined phacoemulsification and pars plana vitrectomy. J Cataract Refract Surg.

