# Supplementary material for: Intraocular lens power calculation formulas accuracy in combined phacovitrectomy: an 8-formulas comparison study
Source: Int J Retina Vitreous. 2021 Aug 18;7:47. doi: 10.1186/s40942-021-00315-7 (PMC8371894; doi:10.1186/s40942-021-00315-7)
Supplement: Supplementary file 1 — Additional file 1: Table S1. Each formula optimised constants. [file 40942_2021_315_MOESM1_ESM.docx]

**Supplemental Table 1** – Each formula optimised constants

| FORMULA | CONSTANTS | Group 1  Phacoemulsification alone  n=100 | Group 2  Combined Phacovitrectomy  n=120 |
| --- | --- | --- | --- |
| KANE | A-Constant | 119.24 | 119.07 |
| PEARL-DGS | A-Constant | 119.36 | 119.16 |
| BARRET UII | A-Constant | 2.08 | 1.98 |
| EVO 2.0 | LF | 119.30 | 119.11 |
| SRK/T | A-constant | 119.33 | 119.15 |
| HOLLADAY 1 | SF | 2.02 | 1.93 |
| HAIGIS | a0 (a1^a^, a2^a^) | -0.53 | -0.63 |
| HOFFER Q | pACD | 5.81 | 5.71 |

LF – lens factor; SF – surgeon factor; pACD – personalized anterior chamber depth. ^a^Haigis values a1 and a2 are from the ULIB website
